# Association Between Vitamin D Deficiency and Cardiometabolic Risk Clustering Among Rural Community-Dwelling Older Adults: A Cross-Sectional Study

**DOI:** 10.3390/healthcare14050627

**Published:** 2026-03-02

**Authors:** HeeJang Yun, Kyeongmin Jang

**Affiliations:** 1 Department of Nursing, Bucheon University, 56 Sosa-ro, Bucheon-si 14774, Gyeonggi-do, Republic of Korea; prof.heejang@mensakorea.org; 2 Department of Nursing, College of Health Sciences, Daejin University, 1007 Hoguk-ro, Pocheon-si 11159, Gyeonggi-do, Republic of Korea

**Keywords:** 25-hydroxyvitamin D, vitamin D deficiency, cardiometabolic risk factor clustering, rural older adults, KNHANES, cross-sectional study

## Abstract

**Background/Objectives**: Cardiometabolic risk factor clustering is common in older adults and is associated with substantially increased cardiometabolic morbidity and mortality. This study aimed to examine the association between serum 25-hydroxyvitamin D [25(OH)D] status and cardiometabolic risk factor clustering among rural community-dwelling older adults. **Methods**: This cross-sectional study analyzed data from the 2022 Korea National Health and Nutrition Examination Survey (KNHANES) for 432 adults aged ≥65 years residing in rural areas. Cardiometabolic risk factor clustering was defined as the presence of ≥2 of the following: abdominal obesity, hypertension, type 2 diabetes mellitus, and dyslipidemia. Multivariable logistic regression was performed adjusting for sociodemographic and behavioral factors. **Results**: Cardiometabolic risk factor clustering was more prevalent among participants with vitamin D deficiency (<15 ng/mL) than among those with sufficient levels (66.0% vs. 44.9%, *p* = 0.006). After adjustment, vitamin D sufficiency (≥15 ng/mL), compared with vitamin D deficiency (<15 ng/mL), was associated with lower odds of clustering (aOR = 0.422, 95% CI: 0.219–0.811, *p* = 0.010). Meeting the WHO physical activity guideline was also associated with lower odds of clustering (AOR = 0.450, 95% CI: 0.226–0.897, *p* = 0.023). **Conclusions**: Lower vitamin D status was associated with a higher prevalence of cardiometabolic risk factor clustering. These findings support the consideration of vitamin D assessment and lifestyle-focused strategies within community health nursing practice to reduce cardiometabolic risk in rural aging populations.

## 1. Introduction

Population aging has accelerated worldwide, accompanied by a growing burden of chronic cardiometabolic conditions in older adults [1]. In South Korea, hypertension remains a major public health concern [2], and the management of hypertension, type 2 diabetes mellitus, and dyslipidemia in older adults represents an important clinical and preventive priority [3]. In particular, hypertension affects a substantial proportion of older adults and is a major contributor to cardiovascular morbidity in later life [2]. These conditions often co-occur, a phenomenon referred to as cardiometabolic risk factor clustering [4]. Such clustering is associated with increased risks of cardiovascular disease, stroke, and all-cause mortality [5]. From a clinical and public health perspective, clustering reflects cardiometabolic multimorbidity rather than isolated conditions [4,5]. Therefore, examining clustering patterns may improve risk stratification and help guide more targeted and integrated prevention and management strategies in older adults, particularly in resource-limited settings. Identifying modifiable factors related to clustering is therefore a key priority for prevention in ageing populations.

Vitamin D has gained attention beyond its classical role in calcium and bone homeostasis. Emerging evidence suggests that vitamin D may influence cardiometabolic regulation through multiple mechanisms, including inflammation, insulin sensitivity, endothelial function, and the renin–angiotensin–aldosterone system [6]. In addition, vitamin D has been proposed to exert vasoprotective effects by modulating endothelial function, including nitric oxide bioavailability and endothelial nitric oxide synthase activity [7].

Because vitamin D is fat-soluble, circulating 25-hydroxyvitamin D [25(OH)D] reflects not only intake and cutaneous synthesis but also distribution and storage in body tissues. Greater adiposity has been associated with lower circulating 25(OH)D through mechanisms such as volumetric dilution and sequestration into adipose tissue [8]. These biologic considerations provide a rationale for examining vitamin D status in relation to cardiometabolic risk factor clustering.

Vitamin D deficiency is common in older adults due to age-related reductions in cutaneous synthesis, limited sunlight exposure, and insufficient dietary intake [9]. Rural older adults may be particularly vulnerable because of seasonal sunlight limitations, reduced mobility, and limited access to healthcare and nutritional resources [9]. Moreover, rural populations often face structural barriers to preventive healthcare, including limited availability of medical facilities and public health resources, which may contribute to delayed detection and suboptimal management of cardiometabolic conditions [10]. As a result, examining cardiometabolic risk factor clustering in rural community-dwelling older adults is particularly important for informing context-specific prevention and intervention strategies [10]. In these settings, community and public health nurses are well positioned to integrate vitamin D assessment with lifestyle counseling and chronic disease risk screening.

Although prior studies have reported inverse associations between serum 25(OH)D and individual metabolic abnormalities such as obesity, insulin resistance, and dyslipidemia [11], findings regarding the aggregation of multiple cardiometabolic risks remain inconclusive. Moreover, much of the existing evidence is derived from urban or clinical populations [12], limiting generalizability to rural community-dwelling older adults.

Given that vitamin D may concurrently influence several cardiometabolic pathways [13], examining its association with cardiometabolic risk factor clustering in rural older adults may provide evidence relevant to targeted prevention strategies in underserved communities. Therefore, this study aimed to examine the association between serum 25(OH)D status and cardiometabolic risk factor clustering among rural community-dwelling older adults. Specifically, we compared the prevalence of clustering (defined as the presence of ≥2 of the following: abdominal obesity, hypertension, type 2 diabetes mellitus, and dyslipidemia) by vitamin D status and evaluated whether vitamin D deficiency was associated with cardiometabolic risk factor clustering after adjustment for sociodemographic and lifestyle covariates.

## 2. Materials and Methods

### 2.1. Study Design and Participants

This study was a cross-sectional secondary analysis of data from the 2022 Korea National Health and Nutrition Examination Survey (KNHANES), a nationally representative survey conducted by the Korea Disease Control and Prevention Agency (KDCA). KNHANES uses a multistage, stratified probability sampling design to represent the non-institutionalized Korean population. Data were collected by trained staff following standardized KNHANES protocols, including health interviews, physical examinations, and laboratory testing conducted in mobile examination centers.

**Figure 1:**
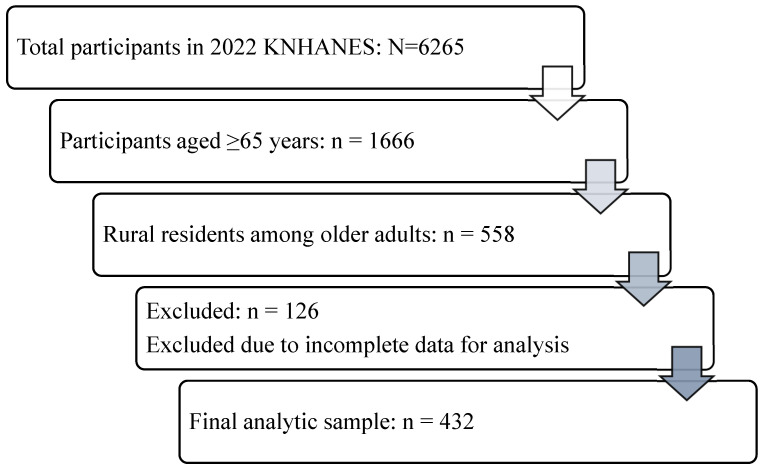
Flow diagram of the study population selection process. Data were obtained from the 2022 Korea National Health and Nutrition Examination Survey (KNHANES). Out of 6265 total participants, 1666 were aged 65 years or older. After selecting 558 rural residents, 126 participants were excluded due to missing data on serum 25(OH)D levels or cardiometabolic risk factors. The final analytic sample consisted of 432 older adults.

Of the 6265 participants surveyed in 2022, 1666 were aged 65 years or older. Among them, 558 individuals were classified as residing in rural areas based on administrative region codes (non-urban areas). Eligible participants for the present analysis were rural residents aged ≥65 years with available data on serum 25-hydroxyvitamin D [25(OH)D], cardiometabolic risk factors, and prespecified covariates. Subsequently, 126 participants were excluded due to missing data on serum 25(OH)D, cardiometabolic risk factors, or key covariates. Consequently, the final analytic sample consisted of 432 rural community-dwelling older adults (Figure 1).

Because this was a secondary analysis of an existing national survey dataset with a fixed number of eligible rural older adults, the analytic sample size was determined by the survey design and the predefined eligibility criteria rather than prospective recruitment. Therefore, an a priori sample size calculation was not used to determine enrollment.

### 2.2. Measures

#### 2.2.1. Cardiometabolic Risk Factor Clustering

The primary outcome was cardiometabolic risk factor clustering, defined as the presence of ≥2 of the following conditions: (1) abdominal obesity (waist circumference ≥90 cm in men or ≥85 cm in women), (2) hypertension (self-reported history of a physician diagnosis from the KNHANES health interview questionnaire), (3) type 2 diabetes mellitus (self-reported history of a physician diagnosis from the KNHANES health interview questionnaire), and (4) dyslipidemia (self-reported history of a physician diagnosis from the KNHANES health interview questionnaire). This definition was based on prior research indicating that co-occurring cardiometabolic conditions are associated with higher risks of cardiovascular events and mortality [4].

#### 2.2.2. Serum Vitamin D

Serum 25-hydroxyvitamin D [25(OH)D] was the primary independent variable and was measured using radioimmunoassay. Participants were categorized as vitamin D deficient if serum 25(OH)D was <15 ng/mL. Because vitamin D status cutoffs differ across guidelines and prior studies, we used <15 ng/mL to represent a more pronounced low 25(OH)D status (moderate-to-severe deficiency), which has been linked to adverse cardiometabolic and cardiovascular outcomes in previous literature [14].

#### 2.2.3. Covariates

Covariates were selected a priori based on their potential associations with both vitamin D status and cardiometabolic risk. Age (years) was treated as a continuous variable, and sex was categorized as male or female. Education was categorized as ≤elementary school or ≥middle school, and household income as the lowest quartile versus all other quartiles. Current smoking status and alcohol use in the past year were coded as yes/no. Physical activity was classified according to the WHO guideline (≥150 min/week of moderate-intensity activity), and participants were categorized as meeting versus not meeting this guideline [15].

### 2.3. Statistical Analysis

Statistical analyses were performed using IBM SPSS Statistics version 29.0 (IBM Corp., Armonk, NY, USA). Participant characteristics were summarized using descriptive statistics. Differences between participants with and without cardiometabolic risk factor clustering were examined using chi-square tests for categorical variables and independent-samples *t*-tests for continuous variables. Normality of continuous variables was assessed using the Shapiro–Wilk test, visual inspection of histograms and Q–Q plots, and evaluation of skewness statistics. All continuous variables demonstrated approximately normal distributions; therefore, independent-samples *t*-tests were used for continuous variables. Associations between vitamin D status and individual cardiometabolic risk factors were assessed using chi-square tests. Multivariable logistic regression analysis was conducted to evaluate the association between vitamin D status and cardiometabolic risk factor clustering after adjusting for age, sex, education, household income, smoking status, alcohol use, and physical activity. Results are presented as adjusted odds ratios (AORs) with 95% confidence intervals (CIs). Covariates were selected a priori based on clinical relevance and previous literature; therefore, no univariable *p*-value screening was used for variable selection, and all prespecified covariates were included in the multivariable model regardless of statistical significance. A two-sided *p* value < 0.05 was considered statistically significant.

## 3. Results

### 3.1. Participant Characteristics by Cardiometabolic Risk Factor Clustering

A total of 432 rural community-dwelling older adults were included in the analysis, of whom 204 (47.2%) had cardiometabolic risk factor clustering (≥2 conditions). As shown in Table 1, participants with clustering had significantly higher mean BMI (25.23 ± 3.36 kg/m^2^ vs. 23.60 ± 2.95 kg/m^2^, *p* < 0.001) and waist circumference (89.47 ± 9.55 cm vs. 84.34 ± 8.08 cm, *p* < 0.001) than those without clustering. Serum 25-hydroxyvitamin D [25(OH)D] concentrations were significantly lower in the clustering group (27.20 ± 10.68 ng/mL vs. 29.36 ± 11.47 ng/mL, *p* = 0.044). The proportion of participants meeting the WHO physical activity guideline was also lower among those with clustering (6.9% vs. 13.2%, *p* = 0.031). No significant differences were observed in age, sex, education, household income, smoking status, or alcohol use in the past year between the two groups (Table 1).

### 3.2. Prevalence of Individual Cardiometabolic Risk Factors According to Serum Vitamin D Levels

As shown in Table 2, vitamin D deficiency (serum 25(OH)D < 15 ng/mL) was associated with a higher prevalence of several cardiometabolic risk factors. Participants with vitamin D deficiency had a significantly higher prevalence of abdominal obesity (61.7% vs. 46.0%, χ^2^ = 11.81, *p* = 0.003) and type 2 diabetes mellitus (40.4% vs. 22.3%, χ^2^ = 7.45, *p* = 0.006) than those with serum 25(OH)D ≥15 ng/mL. The prevalence of overweight/obesity (BMI ≥ 25 kg/m^2^) was also higher in the deficient group (53.2% vs. 38.4%), approaching statistical significance (*p* = 0.051). In contrast, no significant differences were observed for hypertension (*p* = 0.661) or dyslipidemia (*p* = 0.082) by vitamin D status (Table 2).

### 3.3. Clustering of Cardiometabolic Risk Factors by Vitamin D Levels

Cardiometabolic risk factor clustering (≥2 conditions) was more prevalent among participants with vitamin D deficiency. As presented in Table 3, 66.0% of participants with serum 25(OH)D < 15 ng/mL exhibited clustering, compared with 44.9% of those with serum 25(OH)D ≥ 15 ng/mL (χ^2^ = 7.427, *p* = 0.006).

### 3.4. Logistic Regression Predicting Cardiometabolic Risk Factor Clustering

The results of the multivariable logistic regression analysis are presented in Table 4. After adjustment for covariates, three factors were independently associated with lower odds of cardiometabolic risk factor clustering (≥2): meeting the WHO physical activity guideline (AOR = 0.45, 95% CI: 0.226–0.897, *p* = 0.023), alcohol use in the past year (AOR = 0.644, 95% CI: 0.422–0.982, *p* = 0.041), and vitamin D sufficiency (serum 25(OH)D ≥15 ng/mL; reference: <15 ng/mL) (AOR = 0.422, 95% CI: 0.219–0.811, *p* = 0.010). Age showed a borderline association (*p* = 0.055), whereas sex, educational attainment, household income, and smoking status were not significantly associated with clustering. The overall model was statistically significant (χ^2^ = 19.609, df = 8, *p* = 0.012), and the Hosmer–Lemeshow test indicated adequate model fit (χ^2^ = 4.863, df = 8, *p* = 0.772). The model explained 5.9% of the variance in clustering (Nagelkerke R^2^ = 0.059) and correctly classified 58.1% of participants (Table 4).

## 4. Discussion

To our knowledge, few studies have examined the association between vitamin D deficiency and cardiometabolic risk factor clustering specifically among rural community-dwelling older adults in South Korea. In this study, lower serum 25-hydroxyvitamin D [25(OH)D] status was significantly associated with a higher likelihood of having two or more cardiometabolic risk factors. This association remained robust even after adjusting for key sociodemographic and behavioral covariates. These findings add to the growing body of evidence linking vitamin D deficiency to adverse cardiometabolic profiles in aging populations [16,17]. Importantly, our results extend prior research by focusing on rural older adults, a vulnerable group often facing distinctive barriers to maintaining metabolic health—such as limited access to healthcare resources, reduced mobility, and socioeconomic constraints [9,18]. Collectively, our data suggest that low serum 25(OH)D may serve as a valuable biomarker of greater cardiometabolic risk burden in rural aging populations.

Several plausible biological pathways support the observed association between low vitamin D status and cardiometabolic risk clustering. Vitamin D receptors are widely distributed in tissues relevant to metabolic health, including adipose tissue, pancreatic beta-cells, and skeletal muscle. Vitamin D has been implicated in the regulation of systemic inflammation, insulin sensitivity, and lipid metabolism [6,19]. Furthermore, vitamin D deficiency has been linked to endothelial dysfunction and the activation of the renin–angiotensin–aldosterone system (RAAS), mechanisms that may directly contribute to hypertension and vascular dysregulation [20]. Although the cross-sectional nature of our study precludes causal inferences, these physiological mechanisms provide strong biological plausibility for the links observed in this rural older population.

In our analysis of individual risk factors, abdominal obesity and type 2 diabetes were significantly more prevalent among participants with vitamin D deficiency (<15 ng/mL). These findings align with previous observational studies reporting inverse associations between serum 25(OH)D concentrations and adiposity-related indicators as well as glycemic abnormalities [21,22]. Conversely, we did not observe significant differences in the prevalence of hypertension or dyslipidemia based on vitamin D status. This discrepancy may reflect heterogeneity in underlying cardiometabolic phenotypes or the influence of unmeasured confounding factors, such as specific medication use (e.g., statins or antihypertensives), dietary patterns, or seasonal variations in blood pressure, which might attenuate observable associations in population-based samples [23,24]. In addition, self-reported physician diagnosis may reflect heterogeneous disease duration and treatment status, which could further reduce detectable differences by vitamin D status in cross-sectional analyses.

Regarding health behaviors, meeting the WHO physical activity guideline was associated with lower odds of cardiometabolic risk factor clustering in the adjusted model. This reinforces established evidence that regular physical activity is a cornerstone for reducing central adiposity and improving insulin sensitivity [25]. Interestingly, alcohol use in the past year was also inversely associated with clustering in our adjusted model. This finding should be interpreted with caution, as the relationship between alcohol and metabolic health is often J- or U-shaped; while moderate consumption has been linked to favorable metabolic profiles in some cohorts, heavy use is detrimental [26]. Moreover, the present measure captured alcohol use as a binary indicator (yes/no) and did not reflect quantity, frequency, or heavy episodic drinking. Reverse causation is also possible if individuals with cardiometabolic conditions reduced or stopped drinking after diagnosis.

From a healthcare and public health perspective, these findings highlight opportunities for proactive risk identification and integrated prevention strategies in rural communities. Given the high prevalence of both vitamin D deficiency and metabolic risk factors in this demographic, community health practitioners and nurses are well positioned to incorporate vitamin D assessment into routine cardiometabolic risk appraisals. Effective interventions should go beyond simple supplementation; they require a holistic approach that includes counseling on safe sunlight exposure, nutritional education, and the promotion of physical activity [27,28]. Furthermore, integrating these lifestyle-focused strategies into existing community-based chronic disease management programs could be a cost-effective approach to reducing the cardiometabolic burden and promoting healthier aging in underserved rural areas [29,30].

This study has several limitations. First, the cross-sectional design prevents the determination of causality or temporal directionality between vitamin D status and metabolic clustering. Second, cardiometabolic conditions were defined using self-reported physician diagnoses and anthropometric measurements, which may introduce recall bias, although the use of standardized KNHANES protocols mitigates this to some extent. Third, serum 25(OH)D was measured at a single time point, which may not fully reflect long-term status or seasonal variations. Despite these limitations, the study’s strength lies in the use of a nationally representative dataset with biomarker-based assessment of vitamin D, providing valuable insights into a specific and often understudied population—rural older adults.

Future studies using longitudinal designs are needed to clarify temporal relationships between vitamin D status and the development or persistence of cardiometabolic risk factor clustering in rural older adults. Research incorporating repeated 25(OH)D measurements across seasons, as well as more detailed information on dietary intake, supplement use, and medication treatment, may reduce misclassification and better characterize dose–response relationships. In addition, intervention studies that combine strategies to improve vitamin D status with lifestyle modification (e.g., physical activity promotion and nutrition education) could help determine whether such approaches can reduce clustered cardiometabolic risk in rural aging populations.

## 5. Conclusions

In this cross-sectional study of rural community-dwelling older adults in South Korea, vitamin D deficiency (serum 25(OH)D < 15 ng/mL) was independently associated with a significantly higher prevalence of cardiometabolic risk factor clustering. Conversely, maintaining sufficient vitamin D levels and adhering to physical activity guidelines were protective factors. These findings suggest that serum 25(OH)D status should be considered a potential marker for cardiometabolic risk stratification in rural aging populations.

For public health policy and community healthcare practice, these results support the need for integrated intervention programs. Strategies that combine vitamin D screening with lifestyle modifications—specifically targeting physical activity and nutritional support—may be particularly effective in mitigating cardiometabolic multimorbidity. Future prospective studies and randomized controlled trials are warranted to clarify the causal nature of these relationships and to evaluate the clinical efficacy of vitamin D optimization in reducing cardiometabolic risk among older adults in rural settings.

## Figures and Tables

**Table 1:** General characteristics of rural older adults according to vitamin D status (N = 432).

Variable	Total (N = 432) M (SD) or n (%)	VD < 15 (n = 47) M (SD) or n (%)	VD ≥ 15 (n = 385) M (SD) or n (%)	t or χ^2^	*p*
Age (years)	72.57 (5.13)	74.19 (4.90)	72.37 (5.14)	2.26	0.011
Sex (male)	195 (45.1)	22 (46.8)	173 (44.9)	0.06	0.807
Education (≥middle school)	169 (39.1)	12 (25.5)	157 (40.8)	4.08	0.043
Income ≥ Mid-High	221 (51.2)	23 (48.9)	198 (51.4)	0.11	0.747
Current smoker	42 (9.7)	2 (4.3)	40 (10.4)	1.79	0.18
Current drinker (past year)	216 (50.0)	16 (34.0)	200 (51.9)	5.38	0.02
Meets WHO PA guideline	44 (10.2)	3 (6.4)	41 (10.6)	0.83	0.361
BMI (kg/m^2^)	24.37 (3.25)	24.79 (3.52)	24.32 (3.22)	1.36	0.174
Waist circumference (cm)	86.76 (9.16)	88.94 (10.55)	86.49 (8.96)	2.05	0.042
CVD Cluster (yes)	205 (47.5)	31 (66.0)	174 (45.2)	7.43	0.007

Note: Values are presented as mean ± standard deviation (SD) for continuous variables and as number (percentage) for categorical variables. *p* values were calculated using independent-samples *t*-tests or chi-square (χ^2^) tests, as appropriate. Vitamin D deficiency was defined as serum 25(OH)D < 15 ng/mL. Cardiometabolic risk factor clustering was defined as the presence of ≥2 of the following: abdominal obesity (waist circumference ≥90 cm in men or ≥85 cm in women), hypertension, type 2 diabetes mellitus, and dyslipidemia. Abbreviations: BMI, body mass index; M, mean; PA, physical activity; SD, standard deviation; VD, vitamin D; 25(OH)D, 25-hydroxyvitamin D.

**Table 2:** Prevalence of individual cardiometabolic risk factors according to serum vitamin D levels (N = 432).

Variable	Vitamin D < 15 ng/mL (n = 47)	Vitamin D ≥ 15 ng/mL (n = 385)	χ^2^	*p*
BMI ≥ 25 kg/m^2^, No. (%)	25 (53.2)	148 (38.4)	3.8	0.051
High waist circumference, No. (%)	29 (61.7)	177 (46.0)	11.81	0.003
Hypertension, No. (%)	30 (63.8)	233 (60.5)	0.19	0.661
Diabetes, No. (%)	19 (40.4)	86 (22.3)	7.45	0.006
Dyslipidemia, No. (%)	24 (51.1)	146 (37.9)	3.03	0.082

Note. Values are presented as number (percentage). *p* values were calculated using the chi-square (χ^2^) test. Abdominal obesity was defined as a waist circumference ≥90 cm in men or ≥85 cm in women. Abbreviations: BMI, body mass index; VD, vitamin D.

**Table 3:** Clustering of ≥2 cardiometabolic risk factors by vitamin D levels (N = 432).

Vitamin D Status (ng/mL)	Clustering Absent n (%)	Clustering Present n (%)	χ^2^	*p*
<15	16 (34.0)	31 (66.0)	7.427	0.006
≥15	212 (55.1)	173 (44.9)		
Total	228 (52.8)	204 (47.2)		

Note: Values are presented as n (%). *p* values were calculated using the chi-square (χ^2^) test. Cardiometabolic risk clustering was defined as the concurrent presence of ≥2 cardiometabolic risk factors (abdominal obesity, hypertension, diabetes mellitus, or dyslipidemia). Abbreviations: 25(OH)D, 25-hydroxyvitamin D.

**Table 4:** Logistic regression predicting clustering of cardiometabolic risk factors (N = 432).

Variable	B	*p*	aOR	95% CI
Sex (1 = female)	−0.120	0.597	0.887	0.569–1.383
Education (≤elementary)	0.126	0.592	1.134	0.717–1.794
Income (lowest quartile)	−0.051	0.820	0.951	0.614–1.471
Current smoker (yes)	−0.087	0.806	0.917	0.459–1.830
Alcohol consumption (yes)	−0.440	0.041	0.644	0.422–0.982
Meets WHO PA guideline (yes)	−0.798	0.023	0.450	0.226–0.897
Age (years)	−0.042	0.055	0.959	0.918–1.001
Vitamin D ≥ 15 ng/mL (yes)	−0.863	0.010	0.422	0.219–0.811
Constant	4.200	0.022	66.654	—

Note: The model was adjusted for age, sex, education, household income, smoking status, alcohol consumption, and physical activity. Model fit statistics: −2 Log likelihood = 577.936; Cox & Snell R^2^ = 0.044; Nagelkerke R^2^ = 0.059. Hosmer–Lemeshow Test: χ^2^ = 4.863, df = 8, *p* = 0.772. Overall classification accuracy = 58.1%. Abbreviations: aOR, adjusted odds ratio; CI, confidence interval; PA, physical activity; Ref, reference group; WHO, World Health Organization.

## Data Availability

Publicly available datasets were analyzed in this study. This data can be found here: https://knhanes.kdca.go.kr/ (accessed on 20 July 2025).
